# Supplementary material for: Phylogenetic Implication of Large Intergenic Spacers: Insights from a Mitogenomic Comparison of Prosopocoilus Stag Beetles (Coleoptera: Lucanidae)
Source: Animals (Basel). 2022 Jun 21;12(13):1595. doi: 10.3390/ani12131595 (PMC9264858; doi:10.3390/ani12131595)
Supplement: Supplementary file 1 [file animals-12-01595-s001.zip › animals-1739259-supplementary.pdf]

Table S1. The best-fit schemes and evolutionary models for two datasets.

Data	Subset	Model	Partition
Nucleotide	P1	GTR+I+G	atp6_pos1, cytb_pos1
	P2	GTR+I+G	cytb_pos2, cox3_pos2, atp6_pos2, nad3_pos2
	P3	GTR+I+G	cox1_pos3, cox3_pos3, atp6_pos3, cytb_pos3, nad3_pos2, cox2_pos3
	P4	GTR+I+G	atp8_pos2, atp8_pos1, nad2_pos1, nad6_pos1, nad3_pos1
	P5	HKY+I+G	atp8_pos3, nad6_pos3
	P6	GTR+I+G	cox1_pos1, cox3_pos1, cox2_pos1
	P7	GTR+I+G	cox1_pos2, cox2_pos2
	P8	GTR+I+G	nad4l_pos1, nad1_pos1, nad5_pos1, nad4_pos1
	P9	GTR+I+G	nad5_pos2, nad4l_pos2, nad1_pos2, nad4_pos2
	P10	GTR+I+G	nad1_pos3, nad5_pos3, nad4_pos3, nad4l_pos3
	P11	GTR+I+G	nad6_pos2, nad2_pos2
	P12	GTR+G	nad2_pos3
Amino acid	P1	MTREV+I+G	nad2
	P2	MTMAM+I+G	cox1
	P3	MTMAM+I+G	cox2, cytb
	P4	MTREV+I+G	atp8
	P5	MTMAM+I+G	nad4, atp6
	P6	MTREV+I+G	cox3
	P7	MTMAM+I+G	nad3
	P8	MTREV+I+G	nad5
	P9	MTMAM+G	nad4l
	P10	MTMAM+I+G	nad6
	P11	MTREV+I+G	nad1

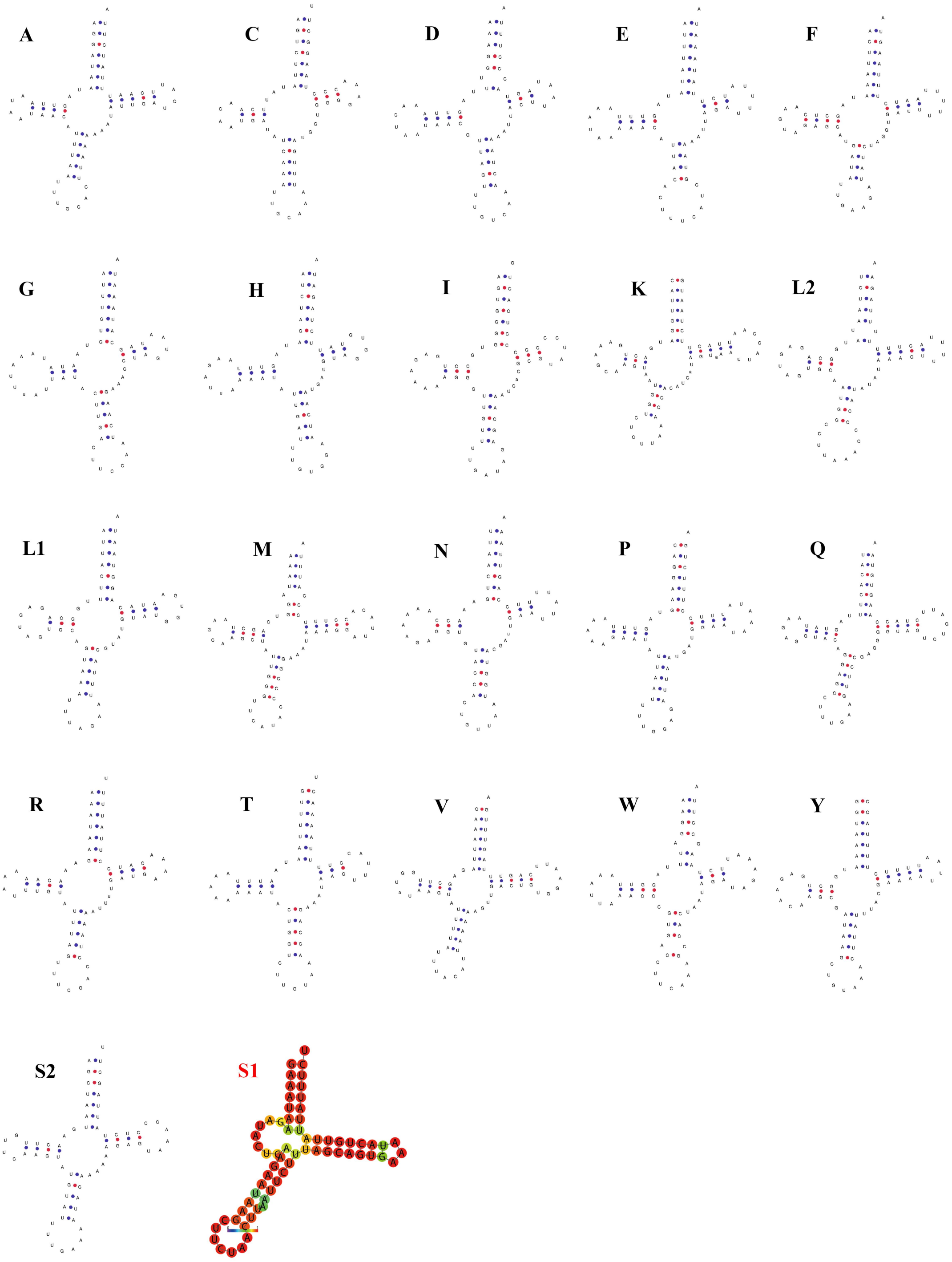

Figure S1. The secondary structure of 22 tRNAs for *P. castaneus*.

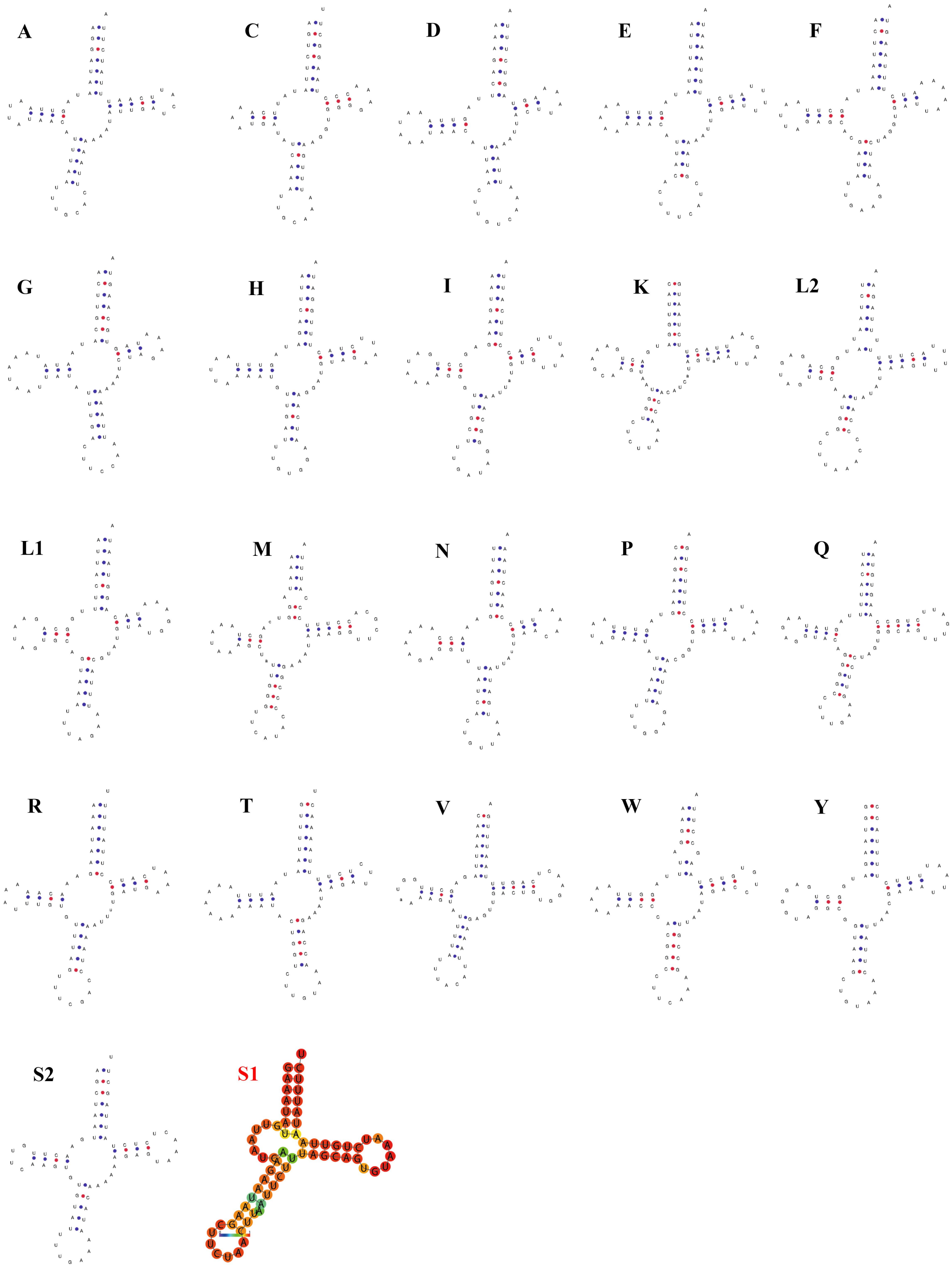

Figure S2. The secondary structure of 22 tRNAs for *P. laterotarsus*.
